# Screening of Probiotic Candidates in Human Oral Bacteria for the Prevention of Dental Disease

**DOI:** 10.1371/journal.pone.0128657

**Published:** 2015-06-08

**Authors:** Tomohiko Terai, Takekazu Okumura, Susumu Imai, Masumi Nakao, Kazuaki Yamaji, Masahiko Ito, Tsuyoshi Nagata, Kimiyuki Kaneko, Kouji Miyazaki, Ayako Okada, Yoshiaki Nomura, Nobuhiro Hanada

**Affiliations:** 1 Yakult Central Institute, Kunitachi, Tokyo, Japan; 2 Department of Translational Research, Tsurumi University School of Dental Medicine, Yokohama, Kanagawa, Japan; Charité, Campus Benjamin Franklin, GERMANY

## Abstract

The oral cavity in healthy subjects has a well-balanced microbiota that consists of more than 700 species. However, a disturbance of this balance, with an increase of harmful microbes and a decrease of beneficial microbes, causes oral disorders such as periodontal disease or dental caries. Nowadays, probiotics are expected to confer oral health benefits by modulating the oral microbiota. This study screened new probiotic candidates with potential oral health benefits and no harmful effects on the oral cavity. We screened 14 lactobacillus strains and 36 streptococcus strains out of 896 oral isolates derived from healthy subjects. These bacteria did not produce volatile sulfur compounds or water-insoluble glucan, had higher antibacterial activity against periodontal bacteria, and had higher adherence activity to oral epithelial cells or salivary-coated hydroxyapatite *in vitro*. We then evaluated the risk of primary cariogenicity and infective endocarditis of the selected oral isolates. As a result, *Lactobacillus crispatus* YIT 12319, *Lactobacillus fermentum* YIT 12320, *Lactobacillus gasseri* YIT 12321, and *Streptococcus mitis* YIT 12322 were selected because they showed no cariogenic potential in an artificial mouth system and a lower risk of experimental infective endocarditis in a rat model. These candidates are expected as new probiotics with potential oral health benefits and no adverse effects on general health.

## Introduction

The oral cavity in healthy subjects has a well-balanced microbiota that consists of approximately 1.0 × 10^11^ microbes/g of dental plaque, and more than 700 species reside on the tongue dorsum, buccal epithelium, hard and soft palates, and other surfaces [1]. Many studies have demonstrated that a disturbance of this balance, with an increase of harmful microbes and a decrease of beneficial microbes, causes oral disorders, such as periodontal disease and dental caries [2]. One of the purposes of oral health is to retain as many teeth as possible, even in the elderly. In fact, the proportion of young adults with dental caries is decreasing and the proportion of the elderly population with many adult teeth is increasing in developed countries because of the establishment of daily dental care for improving oral health [3]. However, the prevalence of periodontal disease has been estimated at approximately 75% and 74% in the USA and Japan, respectively [4,5]. In particular, it has been revealed that periodontal disease not only deteriorates the quality of life but also increases the risk of systemic disorders such as coronary heart disease, cardiovascular disease, cerebrovascular disease, diabetes, pre-term low birth weight babies, preeclampsia, and respiratory infections [6,7]. Therefore, the importance of the prevention of periodontal disease has been recognized in countries where lifestyle-related diseases are increasing in prevalence.

Antibiotics have been used in the treatment of infectious disease including oral disease, however, their use should be restricted because the spread of antibiotic-resistant bacteria has become a serious problem. Another tool that can be used to modulate the microbiota is probiotics, which are defined as living microorganisms that confer a health benefit on the host when administered in a sufficient amount [8]. Lactobacilli and bifidobacteria are typical probiotics that can induce many health benefits such as anti-constipation, anti-diarrhea, anti-infection, anti-carcinogenesis, anti-inflammation, and immune modulation, based on the action of their metabolites and cellular components [9,10]. There is accumulating evidence showing the health benefits of oral probiotics. For example, one or more strains of lactobacilli, streptococci, and/or bifidobacteria isolated from human oral specimens reduce the oral malodor caused by volatile sulfur compounds (VSCs) [11,12] and prevent dental caries [13–16], periodontal disease [17], and other infections such as candidiasis in the human oral cavity [18].

Conversely, the presence of some oral streptococci and lactobacilli is associated with an increased risk of cariogenicity and infectious endocarditis in humans. Mutans streptococci (*Streptococcus sobrinus* and *S*. *mutans*) have the ability to convert sucrose into sticky water-insoluble glucan (WIG), one of the main factors in cariogenicity [19]. Furthermore, they are well known and potent pathogens of dental caries caused by the accumulation of lactic acid in dental plaques after their adherence to the tooth surface. Hence, potent acid producers are considered to have the potential to cause dental caries. However, although oral streptococci such as *S*. *sanguinis*, *S*. *oralis*, and *S*. *salivarius* are reported to produce organic acid, their acid tolerance is weaker than that of mutans streptococci, restricting the contribution of these non-mutans oral streptococci to cariogenicity. Several species of oral streptococci, including these three strains, are isolated frequently from patients with endocarditis [20]; in contrast, lactobacilli are isolated very rarely in such patients [21]. As oral bacteria always invade the blood vessels of the gum in the oral cavity, they are potential causes of bacteremia [22]. Therefore, it is important that the association between oral bacteria and infectious endocarditis is evaluated. The Food and Agriculture Organization/World Health Organization guidelines state that probiotics must have no toxicity *in vitro*, in animal models, and in clinical trials. Oral probiotics have been screened so far by checking their *in vitro* efficacy and attributes, including reduced acid production, no risk of cariogenicity, no potential of propagating antibiotic resistance, and no general toxic parameters [23,24]. However, an evaluation of the risk for infectious endocarditis by oral probiotics has never been reported.

This study aimed to screen new probiotic candidates from oral isolates derived from healthy subjects with potential oral health benefits and no harmful effects by evaluating the lack of production of VSCs and WIG, antibacterial activity against pathogenic bacteria of periodontal disease and dental caries, higher adherence to salivary-coated hydroxylapatite (S-HA) or oral epithelial cells *in vitro*, no cariogenicity in an artificial mouth system (AMS), and a lower risk of experimental infectious endocarditis in a rat model. Four such probiotic strains were successfully selected.

## Materials and Methods

### Subjects

We recruited 56 volunteers (34 men, 22 women; mean age 40.6 ± 11.7 years, range 25–66 years). Volunteers with a smoking habit, dental caries of more than C2, probing pocket depth of more than 4 mm with bleeding on probing, or oral malodor evaluated by the OralChroma (Abimedical Corporation, Kanagawa, Japan) method [25] were excluded. Finally, 32 healthy volunteers (21 men, 11 women; mean age 39.4 ± 10.3 years, range 26–66 years) were enrolled as subjects for the isolation of oral bacteria. The purpose and content of this study were explained fully to all subjects, whose signed informed consent was obtained prior to enrollment. The protocol of this study was specifically approved by the Human Studies Committee of the Yakult Central Institute, Tokyo, Japan, in accordance with the guidelines of the Helsinki Declaration and the Human Studies Committee (approval number: 017).

### Collection of oral specimens and isolation of oral bacteria

Dental plaque and tongue coatings were collected from each subject and were mixed and diluted in an anaerobic transport medium [26]. Immediately after sampling, they were spread on deMan, Rogosa, and Sharpe (MRS) agar plates (Difco; Becton Dickinson, Detroit, MI), *Lactobacillus* selection (LBS) agar plates (Nikken Biomedical Laboratory, Kyoto, Japan), and Mitis-salivarius (MS) agar plates (Difco; Becton Dickinson). These plates were incubated at 37°C for 3 days under anaerobic conditions in a globe box (Anaerobic work station “Concept mini;” GSI Creos, Tokyo, Japan) or an anaerobic jar containing an AnaeroPack (Mitsubishi Gas Chemical, Tokyo, Japan). For colonies with different morphology, 2 or 3 colonies with the same morphology were picked up and cultured in MRS broth or brain heart infusion (BHI) broth (Difco; Becton Dickinson) at 37°C for 24–48 h under the above-mentioned anaerobic conditions. The cultured bacterial cells were collected by centrifugation (1,912 × *g*, 10 min, 4°C), suspended in the stock medium (2-fold concentrated BHI broth and glycerol, 1:1), and frozen at -80°C.

### Production of volatile sulfur compounds

Each oral isolate was pre-cultured in MRS broth at 37°C for 24 h under the above-mentioned anaerobic conditions. Forty microliters of the culture were inoculated into 4 mL Gifu anaerobic medium broth (Nissui, Tokyo, Japan) containing 1% D-glucose and 0.5 mM DL-methionine and cultured at 37°C for 24 h under anaerobic conditions. After the addition of 0.16 mL of 6 M HCl to the culture to reduce the pH below 1, VSCs (hydrogen sulfide, methyl mercaptan, and dimethyl sulfide) in the headspace of each culture were analyzed with an OralChroma portable gas chromatograph according to a previous report [27]. The optical density at 550 nm (OD_550_) of each culture was also measured to calculate the amount of VSCs/mL culture/1 OD_550_. *Fusobacterium nucleatum* YIT 6069^T^, a potent VSC producer [28], was used as a positive control. *Streptococcus mitis* YIT 2035^T^, a low VSC producer, was used as a negative control because *S*. *mitis* is an indigenous oral bacterium in humans and dominant in subjects without oral malodor [29]. Bacteria that produced less VSCs than *S*. *mitis* YIT 2035^T^ were selected.

### Production of water-insoluble glucan

According to a method in a previous report [30], the ability of bacteria to produce WIG was evaluated. Briefly, each oral isolate was pre-cultured in MRS broth or BHI broth at 37°C for 24 h under anaerobic conditions. Forty microliters of the culture were inoculated into 4 mL heart infusion (HI) broth (Difco; Becton Dickinson) or a mixed broth of MRS and HI (7:3, v/v) containing 1% sucrose and cultured at 37°C at a 45° angle under anaerobic conditions. After 24 h, the culture supernatant was removed from the glass tube and 4 mL sterilized phosphate buffered saline (sPBS) were poured slowly along the inner surface of the glass tube. The tube was rotated gently 3 times by hand to wash its inner surface. After removing the sPBS, WIG on the inner surface of the glass tube was evaluated visually, and its presence was used to determine whether the bacterium was a WIG producer. *S*. *mutans* ATCC 25175 and *S*. *sobrinus* ATCC 33478 were employed as positive controls with high WIG producing potential.

### Antibacterial activity against oral pathogenic bacteria

A radial diffusion assay [31] was employed to evaluate antibacterial activity against periodontal pathogens, namely *Porphyromonas gingivalis* ATCC 33277, *Prevotella intermedia* ATCC 25611, and *Aggregatibacter actinomycetemcomitans* Y4, also known as ATCC 43718, and cariogenic pathogens, namely *S*. *mutans* ATCC 25175 and *S*. *sobrinus* ATCC 33478. These bacteria were maintained at Tsurumi University. *P*. *gingivalis* ATCC 33277 and *P*. *intermedia* ATCC 25611 were pre-cultured in tryptic soy (TS) broth (Difco; Becton Dickinson) supplemented with 5 μg/mL hemin (Wako Pure Chemical Industries Ltd.) and 0.5 μg/mL menadione (Wako Pure Chemical Industries Ltd.), while the other pathogenic bacteria were pre-cultured in BHI broth at 37°C for 24 h under anaerobic conditions. The culture supernatant of the pre-culture for 24 h of each oral isolate obtained by centrifugation under the above conditions was filtered through a 0.22-μm membrane filter (Merck Millipore, Billerica, MA). In this screening, both the isolates with lower antibacterial activity and the isolates with lower growth potential were excluded by adopting the same incubation time of 24 h for each oral isolate. TS agar medium, composed of 6 mg TS, 2 μL Tween 20 (Wako Pure Chemical Industries Ltd., Osaka, Japan), and 100 mg agarose (Wako Pure Chemical Industries Ltd.) in 10 mL deionized water, and 0.1 mL of the culture of each pathogenic bacterium were mixed sufficiently to prepare an agar plate. The agar plate was punched to make a 2.5 mm diameter well, into which 5 μL of the filtrate of each oral isolate culture were added and pre-incubated at 37°C for 60 min. Ten milliliters of TS agar medium without Tween 20 were poured on the agar plate to prepare the overlaid agar plate, which was incubated at 37°C for 18–48 h. When *P*. *gingivalis* ATCC 33277 or *P*. *intermedia* ATCC 25611 was used as a target, 5 μg/mL hemin and 0.5 μg/mL menadione were added to the TS medium. As a positive control, 82.5 U/mL bacitracin (Wako Pure Chemical Industries Ltd.) and 0.05–0.2 mg/mL tetracycline-HCl (Wako Pure Chemical Industries Ltd.) were employed for cariogenic pathogens of streptococci and other periodontal disease pathogens, respectively. Antibacterial activity was determined by the diameter of the inhibited zone before and after adjustment of the filtrate of each oral isolate culture with a 2 N NaOH solution to pH 7 (neutralization).

### Adherence activity to salivary-coated hydroxyapatite

To evaluate retention ability in the oral cavity, the ability of the bacteria to adhere to S-HA was measured by a previously reported method [32]. Briefly, human saliva was filtered through a 0.22-μm filter (Merck Millipore) after being heated at 60°C for 30 min and centrifuged (10,000 × *g*, 10 min, 4°C), and S-HA beads were prepared by incubating HA beads (ceramic hydroxyapatite; Bio-Rad Laboratories, Inc., Hercules, CA) in sterilized human saliva at 37°C for 30 min with shaking. The bacterial cells of each oral isolate, pre-cultured in MRS broth under the above conditions, were collected and washed twice in sPBS by centrifugation, and they were then re-suspended in sPBS to adjust the OD_550_ to 1. Five milligrams of S-HA beads and 2 mL of the bacterial cell suspension were incubated at 37°C for 60 min with shaking. After the test tube was left for 10 min for the S-HA beads to settle, 1 mL of the collected supernatant was mixed vigorously with 0.1 mL of a 0.1 M EDTA solution to dissolve the remaining HA particles. Both the OD_550_ of the mixture and the control containing the bacterial cell suspension alone were measured. The adherence rate (%) to the S-HA beads was calculated using the following formula:

$$\text{Adherence rate}\left(\text{\%}\right)\text{=}\left(\left[{\text{OD}}_{550}{\text{of control-OD}}_{\text{550}}\text{of sample}\right]{/\text{OD}}_{\text{550}}\text{of control}\right)\times 100$$

Its adherence rate was used to determine whether the bacterium was adherent to S-HA.

### Adherence to oral epithelial cells

To evaluate the retention ability of the bacteria in the oral cavity, bacterial adherence to oral epithelial cells was measured. Oral epithelial cells, JCRB0831 HO-1-N-1 (HO) cells and JCRB0623 HSC-3 (HSC) cells, which originated from human buccal mucosa carcinoma and human tongue carcinoma, respectively, were obtained from the JCRB cell bank (National Institute of Biomedical Innovation, Osaka, Japan). HO and HSC cells were pre-cultured in Dulbecco’s modified Eagle’s medium (D-MEM; Gibco Life Technologies, Carlsbad, CA) and D-MEM/F12 medium (Gibco Life Technologies), respectively, containing 10% calf serum at 37°C under 5% CO_2_ + 95% humidified air. The cells were seeded in an 8-well Lab-Tek II chamber slide (Nunc Thermo Fisher Scientific, Rockford, IL) at 2.0 × 10^4^ cells/well in 0.2 mL medium, cultured for 48–96 h under the above conditions, and washed 3 times in Roswell Park Memorial Institute (RPMI) 1640 medium (Gibco Life Technologies) to remove non-adhering cells. The bacterial cells of each oral isolate, pre-cultured in MRS broth, collected, and washed in sPBS under the above conditions, were suspended in sPBS and the OD_660_ was adjusted to 0.25. The suspension was added at 0.2 mL/well and incubated at 37°C for 10 min. After washing the chamber slide in RPMI 1640 medium 3 times to remove non-adhering bacteria, the adhering bacteria were visualized after Gram staining. Thereafter, the number of adhering bacteria per 0.16 mm^2^ of the sheet of oral epithelial cells was counted and averaged for 6 different fields per well under a microscope (BX50; Olympus Corporation, Tokyo, Japan). The obtained value was converted into cell count per mm^2^.

### Experimental infective endocarditis

To assess the safety of the oral isolates when they invade blood vessels, the risk of infective endocarditis from the oral isolates was evaluated in a pre-clinical experiment using rats, according to previous reports [33,34]. Isolated bacterial cells cultured in MRS broth, collected, and washed in sterilized saline under the above conditions, were suspended in sterilized saline to make 4.0–9.0 × 10^6^ colony forming units (CFU)/mL. *L*. *rhamnosus* YIT 0227 (PHLS A103/70), a clinical isolate and an inducer of experimental infective endocarditis [35], was employed as a positive control. The protocol of this study was specifically approved by the Ethical Committee for Animal Experiments of Yakult Central Institute (approval numbers: 08–0229 and 08–0280). Male SD (Crl:CD) rats at 8 weeks of age were obtained from Charles River Laboratories (Kanagawa, Japan) and maintained and treated in accordance with the guidelines of the Ethical Committee for Animal Experiments of Yakult Central Institute. All efforts were made to minimize suffering. Under anesthesia with a ketamine-xylazine drug mixture (18:5), the external jugular artery on the right side of the neck was exposed. A polyethylene catheter with an external diameter of 0.61 mm and a length of approximately 20 cm (SP10; Natsume Seisakusho, Tokyo, Japan) was passed down the artery to the left ventricle of the heart and placed to induce experimental infective endocarditis. The next day, each suspension of bacterial cells was injected via the trail vein at 2.0–5.0 × 10^6^ CFU/0.5 mL/rat for 6–7 rats per group. After 4 days, venous blood was collected under anesthesia with Nembutal (Abbott Laboratories, Chicago, IL) and diluted in sterilized saline and then spread on an MRS agar plate. After the rats were sacrificed by bleeding, vegetation at the heart valve was removed, weighed, homogenized, diluted in sterilized saline, and spread on an MRS agar plate. After incubation at 37°C for 3 days, CFU/mL of the blood and vegetation in each sample were determined. Inducing potential was judged as positive when the bacteria administered were detected from either the blood or vegetation in more than 2 rats per group.

### Potential of primary cariogenicity

To evaluate the potential of primary cariogenicity, demineralization of bovine tooth enamel and the production of WIG on the enamel were examined in an AMS according to a previous report [36]. The bacterial cells of each oral isolate, pre-cultured in MRS broth, collected, and washed in sPBS under the above conditions, were suspended in sPBS to adjust the OD_540_ to 1. *S*. *sobrinus* 6715, maintained at Tsurumi University and available as ATCC 27351, was pre-cultured in TS broth at 37°C for 16 h under anaerobic conditions. The AMS consisted of 3 chambers, and 4 bovine enamel slabs were placed on a Teflon holder around the bulb of a flat pH electrode in each chamber. Each bacterial suspension and nutrient medium (MRS/TS) containing 2.5% sucrose (final concentration 1% on the flat pH electrode) were dropped continuously on the bovine enamel slabs and incubated at 37°C for 20 h to form a biofilm, while the pH underneath the artificial biofilm that formed on the flat bulb of the pH electrode was monitored with a multiple pH recorder. After 20 h, enamel slabs and flat bulb were sufficiently washed with sPBS to remove cell-free glucan, water-soluble glucan and the added sucrose. The biofilm that formed on enamel slabs and flat bulb was suspended in a 0.5 N NaOH solution to dissolve the WIG produced in the biofilm. The suspension was centrifuged (1,912 × *g*, 20 min, 4°C) to separate WIG from the bacterial cells. The amount of WIG in the NaOH solution was quantified colorimetrically by the phenol-sulfuric acid method. Moreover, the Vicker’s hardness values of the enamel slabs were determined and compared before and after incubation. The differences in hardness (Δgf) were used to infer the degree of demineralization.

### Identification of isolates based on 16S rDNA partial sequencing

DNA was extracted using the benzyl-chloride method [37]. Cell pellets of each isolate were collected from each culture by centrifugation (1,912 × *g*, 10 min, 4°C). The pellets were re-suspended in 200 μL extraction buffer (100 mM Tris-HCl, 40 mM EDTA; pH 9.0). Glass beads (diameter, 0.1 mm; 300 mg), in 400 μL benzyl chloride, were added to each resuspension and mixed vigorously for 30 s using a FastPrep-24 (M.P. Biomedicals, Irvine, CA) at a power level of 6.5. After mixing, 50 μL of 10% sodium dodecyl sulfate were added and incubated at 50°C for 20 min, and 150 μL of 3 M sodium acetate were added to each sample, which was then cooled on ice for 10 min. After centrifugation (20,630 × *g*, 5 min, 4°C), the supernatant was collected. DNA was precipitated with isopropanol and washed with 70% ethanol. Finally, each DNA pellet was diluted in 60 μL TE buffer (10 mM Tris-HCl, 1 mM EDTA; pH 8).

Isolates were identified at the species level through polymerase chain reaction (PCR) sequencing of the 16S rRNA gene using the universal primers 63F (5′-GCYTAAYACATGCAAGTMG-3′) and 15R (5′-AAGGAGGTGATCCARCCGCA-3′) [38]. PCR was carried out in a 35 μL reaction volume containing 3.2 μL of 10× PCR buffer (1× PCR buffer: 10 mM Tris-HCl [pH 8.3], 50 mM KCl, 1.5 mM MgCl_2_,), 200 μM each dNTP, 0.5 U Ex Taq HS DNA polymerase (TAKARA, Shiga, Japan), 0.4 μM of each respective primer, and 10 ng DNA template. The PCR amplification program consisted of an initial heating step at 94°C for 20 s, 30 cycles at 94°C for 20 s, 55°C for 20 s, and 72°C for 20 s, and a final extension step at 72°C for 3 min. All amplifications were performed on a DNA Engine Thermal Cycler (Bio-Rad Laboratories, Inc.). Amplicons were purified using a Montage PCR Centrifugal Filter Device (Merck Millipore) and sequenced using the primers 63F (5′-GCYTAAYACATGCAAGTMG-3′) and 520R (5′-ACCGCGGCTGCTGGC-3′) [39], and BigDye Terminator v1.1 Chemistry (Life Technologies) on a 3130xl Genetic Analyzer (Life Technologies). The resulting sequences were used to search the DDBJ database using the BLAST algorithm (http://blast.ddbj.nig.ac.jp/blast/blastn?lang=jp), and the isolates were identified on the basis of the highest scores.

## Results

### Number of isolated bacteria

**Fig 1:**
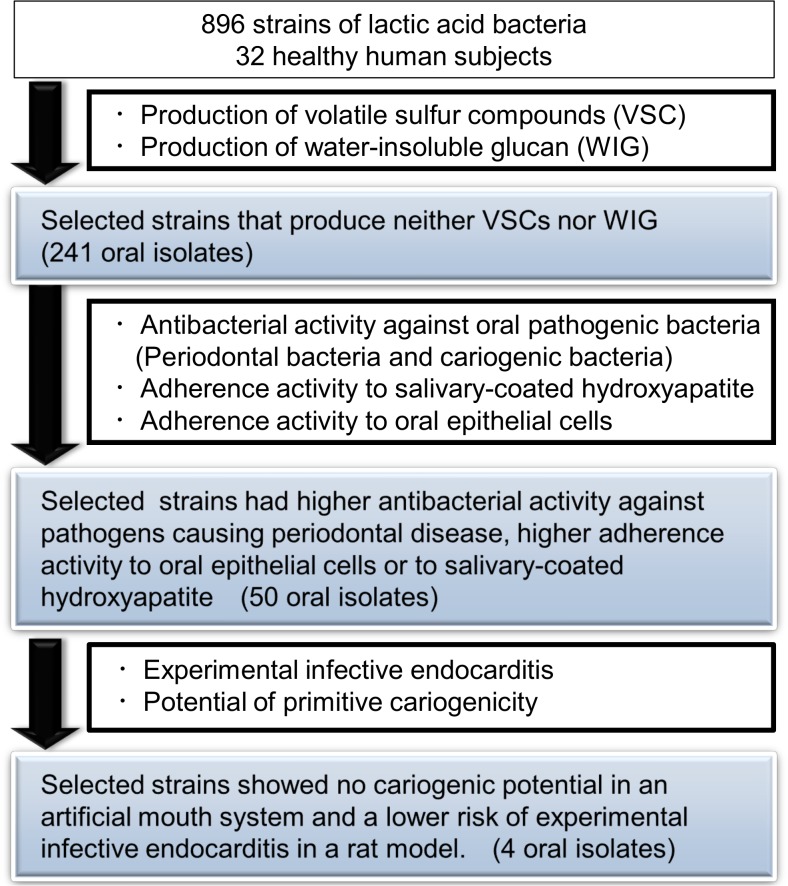
Screening of human oral probiotic candidates with potential oral health benefits and no harmful effects.

A total of 896 oral isolates were obtained from 32 healthy subjects. They consisted of 350 isolates from MS agar plates, 376 isolates from MRS agar plates, and 170 isolates from LBS agar plates, and deposited as an oral bacterial library to apply the following screening steps (Fig 1).

### Production of volatile sulfur compounds and water-insoluble glucan

*F*. *nucleatum* YIT 6069^T^, as a positive control, produced a high amount of VSCs (hydrogen sulfide, 929 μg/10 mL culture/1 OD_550_; methyl mercaptan, 7.02 mg/10 mL culture/1 OD_550_). In contrast, *S*. *mitis* YIT 2035^T^ produced much less VSCs (hydrogen sulfide, 0.7 μg/10 mL culture/1 OD_550_; methyl mercaptan, 1.2 μg/10 mL culture/1 OD_550_). Dimethyl sulfide was below the detection limit for the controls and all isolates. As shown in Table 1, 391 oral isolates in the library had higher VSC production than *S*. *mitis* YIT 2035^T^. In addition, 146 oral isolates were non-producers and 359 oral isolates were lower producers.

**Table 1:** Ability of oral isolates to produce VSCs and WIG.

		WIG production^b^			
		−	+/−	+	Total
**VSC production** ^**a**^	**−**	22	47	77	146
	**+/−**	72	100	187	359
	**+**	82	125	184	391
**Total**		176	272	448	896

^a^VSC (volatile sulfur compound) production *in vitro* was evaluated with an OralChroma. −, < detection limit; +/−, produced H_2_S < 0.7 μg/10 mL/1 OD_550_ or CH_3_SH < 1.17 μg/10 mL/1 OD_550_; +, produced H_2_S > 0.7 μg/10 mL/1 OD_550_ or CH_3_SH > 1.17 μg/10 mL/1 OD_550_; (CH_3_)_2_SH was below the detection limit in all strains.

^b^WIG (water-insoluble glucan) production *in vitro*: −, non-producer; +/−, attachment of bacterial cells; +, producer.

*S*. *mutans* ATCC 25175 and *S*. *sobrinus* ATCC 33478, recognized as cariogenic pathogens, produced a remarkable amount of WIG. As shown in Table 1, 448 oral isolates were producers of WIG (+). In addition, 172 oral isolates (-) and 272 oral isolates (+/-) were non-producers and pseudo-producers of WIG, respectively. By using this screening approach, 241 (22 (WIG(-):VSC(-)), 47 (WIG(-):VSC(+/-)), 72 (WIG(+/-):VSC(-)), and 100 (WIG(+/-):VSC(+/-))) oral isolates, which were neither VSC nor WIG producers, were screened initially from the library.

### Identification based on 16S rDNA partial sequencing

Table 2 shows the classification of the screened 241 oral isolates identified using 16S rDNA partial sequencing. The isolates consisted of 6 genera and 29 species. Several species that are known to be associated with disease (e.g., *S*. *pneumoniae* causes pneumonia and *S*. *anginosus* causes aspiration pneumonitis) were excluded. We then applied 206 isolates (62 lactobacillus isolates and 144 streptococcus isolates) of oral lactic acid-producing bacteria (LAB) to the following screening step.

**Table 2:** Identification of oral isolates based on 16S-rDNA partial sequencing.

Genus and species	Number of strains
*Streptococcus salivarius*	38
*Streptococcus oralis*	26
*Streptococcus sanguinis*	24
*Streptococcus mitis*	22
*Streptococcus parasanguinis*	20
*Streptococcus anginosus*	7
*Streptococcus* sp.	7
*Streptococcus cristatus*	5
*Streptococcus infantis*	2
*Streptococcus pneumoniae*	2
*Streptococcus constellatus*	1
*Streptococcus gordonii*	1
*Streptococcus intermedius*	1
*Streptococcus vestibularis*	1
*Lactobacillus gasseri*	31
*Lactobacillus fermentum*	16
*Lactobacillus casei*	5
*Lactobacillus crispatus*	5
*Lactobacillus salivarius*	2
*Lactobacillus vaginalis*	2
*Lactobacillus mucosae*	1
*Lactobacillus oris*	1
*Lactobacillus ultunensis*	1
*Actinomyces* sp.	7
*Actinomyces odontolyticus*	1
*Alloscardovia omnicolens*	1
*Veillonella dispar*	5
*Veillonella atypica*	1
*Bifidobacterium dentium*	3

### Antibacterial activity against oral pathogenic bacteria

**Fig 2:**
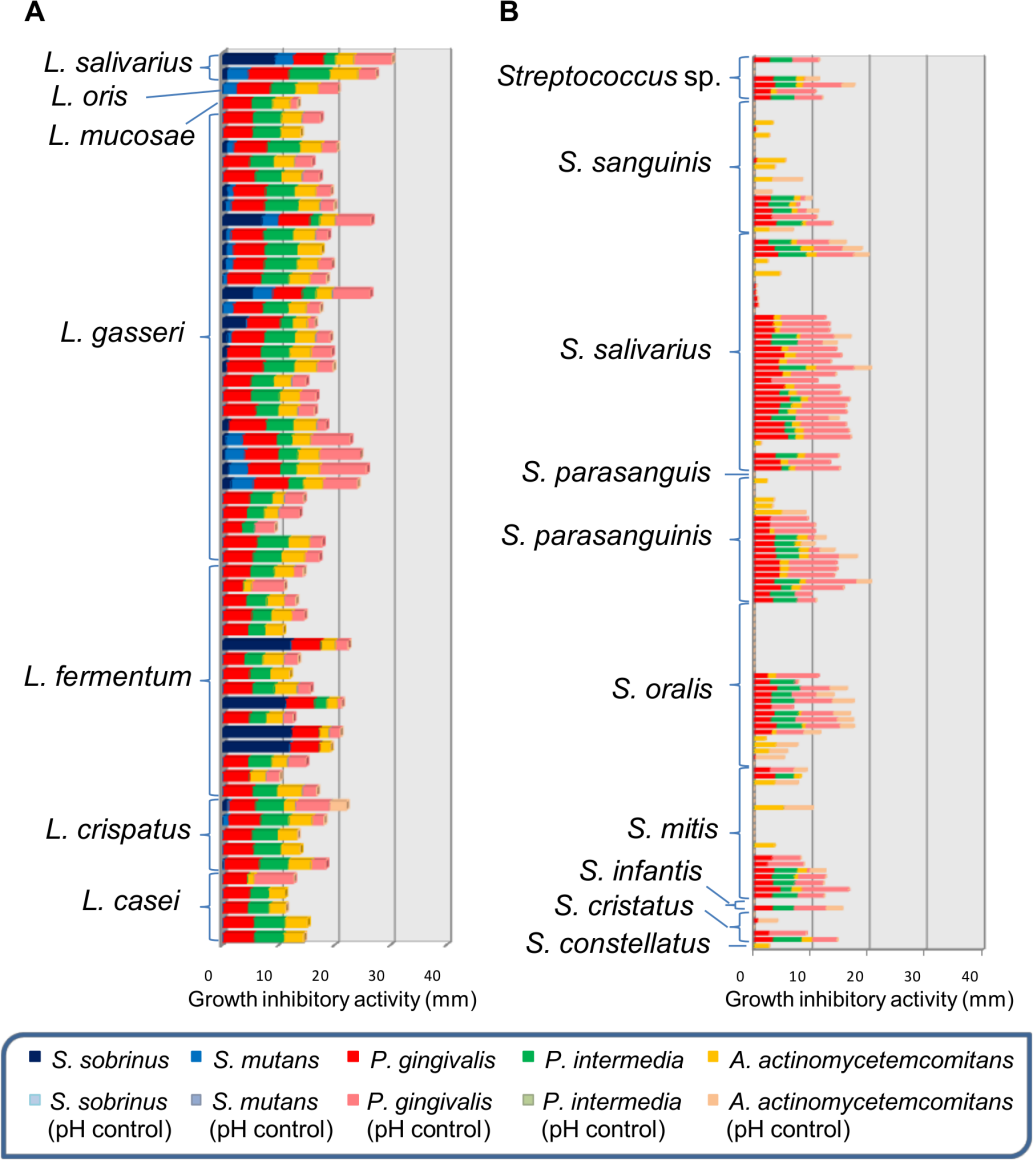
Antibacterial activity of oral LAB isolates against five oral pathogenic bacteria. Antibacterial activity of 206 LAB ([A] 62 lactobacilli and [B] 144 streptococci) isolates was evaluated by the radial diffusion assay. The diameter of the inhibited zone was determined before and after neutralization of the filtrate of each isolate culture.

Fig 2 shows the antibacterial activity of the culture supernatants of 206 LAB oral isolates against 5 oral pathogenic bacteria. Antibacterial activity against *P*. *gingivalis* ATCC 33277 was observed in all lactobacillus isolates before neutralization and most lactobacillus isolates also showed activity after neutralization. Fifty-six lactobacillus isolates showed antibacterial activity against *P*. *intermedia* ATCC 25611, but no strain showed antibacterial activity after neutralization. Similarly, 61 lactobacillus isolates showed antibacterial activity against *A*. *actinomycetemcomitans* Y4; however, only *L*. *crispatus* YIT 12319 still showed antibacterial activity against *A*. *actinomycetemcomitans* Y4 after neutralization. Moreover, antibacterial activity against *S*. *sobrinus* ATCC 33478 and *S*. *mutans* ATCC 25175 was detected in 23 and 21 lactobacillus isolates, respectively, before neutralization, but not after neutralization.

Antibacterial activity against *P*. *gingivalis* ATCC 33277 was found in 80 and 70 streptococcus isolates before and after neutralization, respectively. Forty-seven and 69 streptococcus isolates showed antibacterial activity against *P*. *intermedia* ATCC 25611 and *A*. *actinomycetemcomitans* Y4 before neutralization, while zero and 36 streptococcus isolates showed activity after neutralization, respectively. No streptococcus isolate showed activity against *S*. *mutans* and *S*. *sobrinus* before and after neutralization. Meanwhile, the culture supernatants of oral lactobacilli and streptococci had a pH of 3.8–5.6 and 4.2–6.5, respectively.

### Adherence to salivary-coated hydroxylapatite

Table 3 shows the adherence of 206 oral LAB isolates to S-HA. One hundred and nine streptococcus isolates and 19 lactobacillus isolates adhered to S-HA (+). Most isolates of *S*. *salivarius* and *S*. *oralis* demonstrated adherence to S-HA. The number of lactobacilli which adhered to S-HA was less than that of streptococci. Due to cellular aggregation in the reaction mixture, 4 isolates of *L*. *crispatus* and 1 isolate of *L*. *fermentum* could not be evaluated.

**Table 3:** Adherence of oral LAB isolates to salivary-coated hydroxyapatite.

Genus and species	Number of test strains	Adherence activity to S-HA^a^	
		**+** ^**b**^	**-** ^**c**^
*S*. *salivarius*	38	31	7
*S*. *oralis*	26	23	3
*S*. *parasanguinis*	20	16	4
*S*. *sanguinis*	24	19	5
*S*. *mitis*	22	13	9
*Streptococcus* sp.	7	3	4
*S*. *cristatus*	5	3	2
*S*. *infantis*	2	1	1
*L*. *fermentum*	16	8	8
*L*. *gasseri*	31	8	23
*L*. *casei*	5	3	2
*L*. *salivarius*	2	0	2
*L*. *crispatus*	5	0	5
*L*. *mucosae*	1	0	0
*L*. *oris*	1	0	0
*L*. *ultunensis*	1	0	1

The adherence of 206 LAB (62 lactobacilli and 144 streptococci) isolates was evaluated *in vitro*.

^a^The number of strains adhesion to salivary-coated hydroxyapatite (S-HA).

^b^+; Adherence rate >0%.

^c^-; Includes unmeasurable samples.

### Adherence to oral epithelial cells

**Fig 3:**
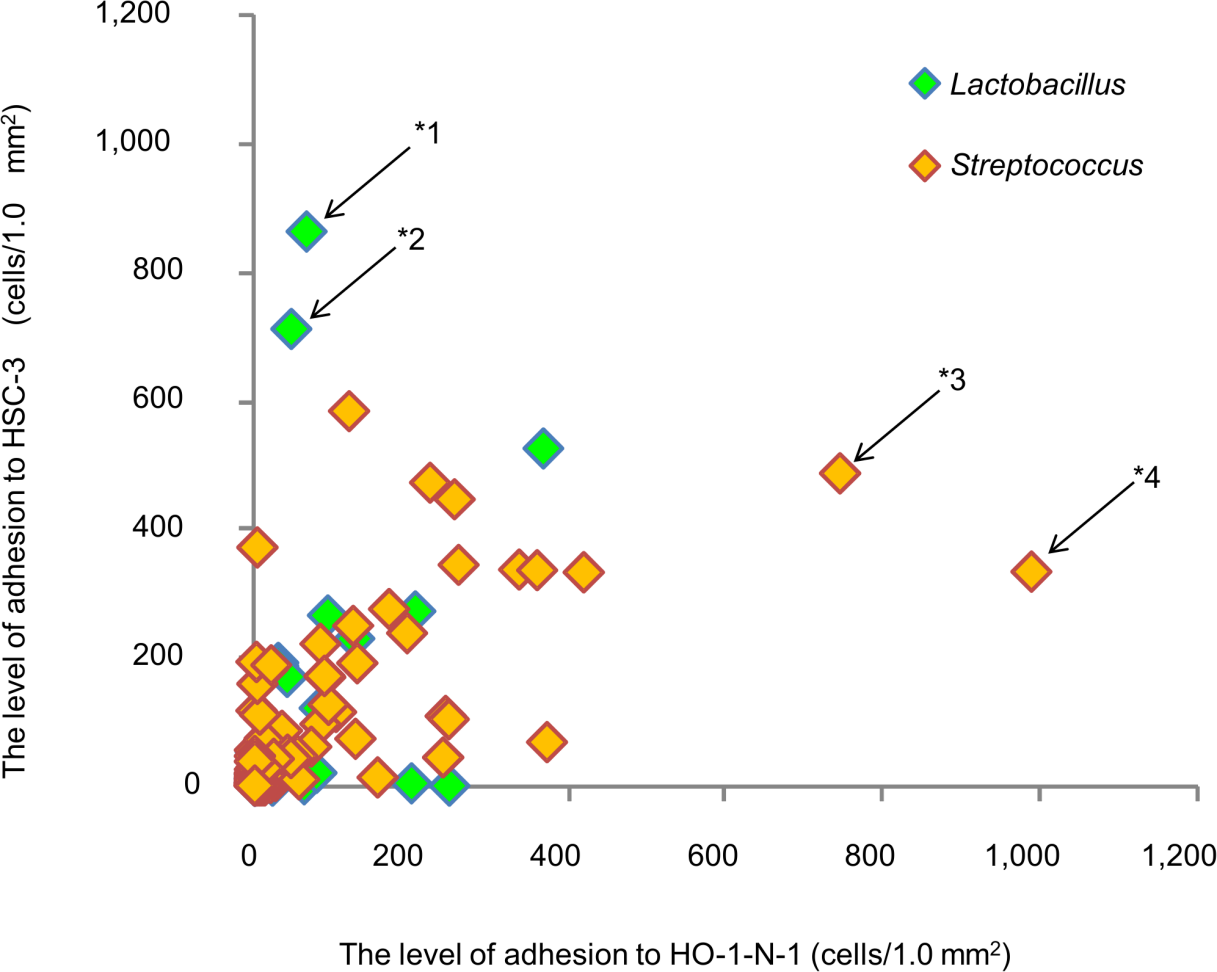
Adherence of oral LAB isolates to two different human oral epithelial cells. The adherence of 206 LAB (62 lactobacilli and 144 streptococci) isolates was evaluated on cell sheets of HO and HSC cells derived from human buccal and tongue tissue, respectively, i*n vitro*. The number of adhering cells was averaged for 6 different fields per well. *L*. *crispatus* YIT 12319 (*1) and *L*. *fermentum* LBS 17–31 (*2) show higher adherence activity to HSC cells. *S*. *mitis* MS 21–11 (*3) and *S*. *salivarius* MS 07–22 show higher adherence activity to HO cells.

Fig 3 shows the adherence of 206 oral LAB isolates to human oral squamous cell carcinoma, that is, HO and HSC cells. As a result, 17 streptococcus isolates and 7 lactobacillus isolates demonstrated adherence, showing more than 60 bacterial cells/mm^2^ for HO and/or HSC cells. There were 3 types of isolates showing similar levels of adherence to both HSC and HO cells, specific adherence to HSC cells by lactobacilli, and specific adherence to HO cells by streptococci. Especially, higher adherence activity with more than 600 bacterial cells/mm^2^ was observed for *L*. *crispatus* YIT 12319, *L*. *crispatus* LBS 17–11, *S*. *mitis* MS 06–32, *S*. *mitis* MS 09–51, *S*. *salivarius* MRS 09–71, *S*. *salivarius* MS 06–12, and *S*. *salivarius* MS 07–22. Consequently, 4 lactobacillus isolates, showing both higher antibacterial activity against oral pathogenic bacteria and higher adherence to oral epithelial cells, and 10 isolates of streptococci, showing higher antibacterial activity against oral pathogenic bacteria and higher adherence to S-HA, were selected and applied to the next screening step.

### Experimental infective endocarditis

Table 4 shows the results of the intravenous administration of 14 oral LAB isolates and a positive control regarding their potential to induce experimental infective endocarditis in a rat model. *L*. *rhamnosus* YIT 0227, adopted as a positive control, was detected in the blood of 5 rats and vegetation of 100% (6/6) rats, showing an average of 8.4 CFU/mL blood and 4.7 × 10^4^ CFU/g vegetation. Therefore, this strain was judged as a potent inducer of infective endocarditis in rats. Four lactobacillus isolates were not detected in the blood of any rat and their levels were below the detection limit in the vegetation, suggesting that they had a low risk for infective endocarditis. In contrast, 9 streptococcus isolates were detected in the blood or vegetation of more than 2 rats of the 2–7 rats examined, suggesting they had a risk for infective endocarditis. However, *S*. *mitis* YIT 12322 was not detected in the blood and was only detected in the vegetation of 1 of the 5 rats examined, suggesting a lower risk. As a result, 5 oral isolates, *L*. *crispatus* YIT 12319, *L*. *crispatus* LBS 17–11, *L*. *fermentum* YIT 12320, *L*. *gasseri* YIT 12321, and *S*. *mitis* YIT 12322, were screened further.

**Table 4:** Potential to induce experimental infective endocarditis of oral LAB isolates.

Strain No.	Detection number (detection rate)		Bacterial number (CFU/g/mL^a^)		Judgment
	Blood	Vegetation	Blood	Vegetation	
*L*. *rhamnosus* YIT 0227^b^	5/6 (83%)	6/6 (100%)	8.4	4.7 × 10^4^	Positive
*L*. *crispatus* YIT 12319	0/4 (0%)	0/4 (0%)	0	< 3.5^c^	Negative
*L*. *crispatus* LBS 17–11	0/5 (0%)	0/5 (0%)	0	<3.5^c^	Negative
*L*. *fermentum* YIT 12320	0/6 (0%)	0/6 (0%)	0	<3.5^c^	Negative
*L*. *gasseri* YIT 12321	0/5 (0%)	0/5 (0%)	0	<3.5^c^	Negative
*S*. *infantis* MRS 20–31	1/3 (33%)	6/7 (86%)	33	4.7 × 10^5^	Positive
*S*. *mitis* YIT 12322	0/5 (0%)	1/5 (20%)	0	8.5 × 10^2^	Negative
*S*. *mitis* MRS 08–21	3/6 (50%)	6/6 (100%)	9.3	2.7 × 10^6^	Positive
*S*. *mitis* MRS 09–41	5/5 (100%)	5/5 (100%)	70	1.2 × 10^6^	Positive
*S*. *oralis* MRS 19–81	0/5 (0%)	5/5 (100%)	0	2.9 × 10^5^	Positive
*S*. *salivarius* MRS 09–71	5/5 (100%)	5/5 (100%)	7.0 × 10^2^	1.2 × 10^6^	Positive
*S*. *salivarius* MRS 49–32	2/2 (100%)	2/2 (100%)	－	9.2 × 10^4^	Positive
*S*. *salivarius* MS 07–22	1/1 (100%)	7/7 (100%)	1.5 × 10^3^	8.4 × 10^6^	Positive
*S*. *salivarius* MS 10–11	3/3 (100%)	3/3 (100%)	7.9 × 10^2^	1.8 × 10^6^	Positive
*S*. *salivarius* MRS 18–31	5/5 (100%)	5/5 (100%)	1.5 × 10^2^	6.2 × 10^4^	Positive

The potential to induce experimental infective endocarditis of 14 LAB (4 lactobacilli and 10 streptococci) isolates was evaluated in a rat model. The inducing potential was judged as positive when the bacteria administered were detected in either the blood or vegetation in more than 2 rats per group (6–7 rats). Rows in which there are less than 6 rats in a group show death due to either a technical mishap or infection during the experiment.

^a^Averaged value of the number of detected bacteria.

^b^Positive control.

^c^Below the detection limit.

### Potential of primary cariogenicity

**Fig 4:**
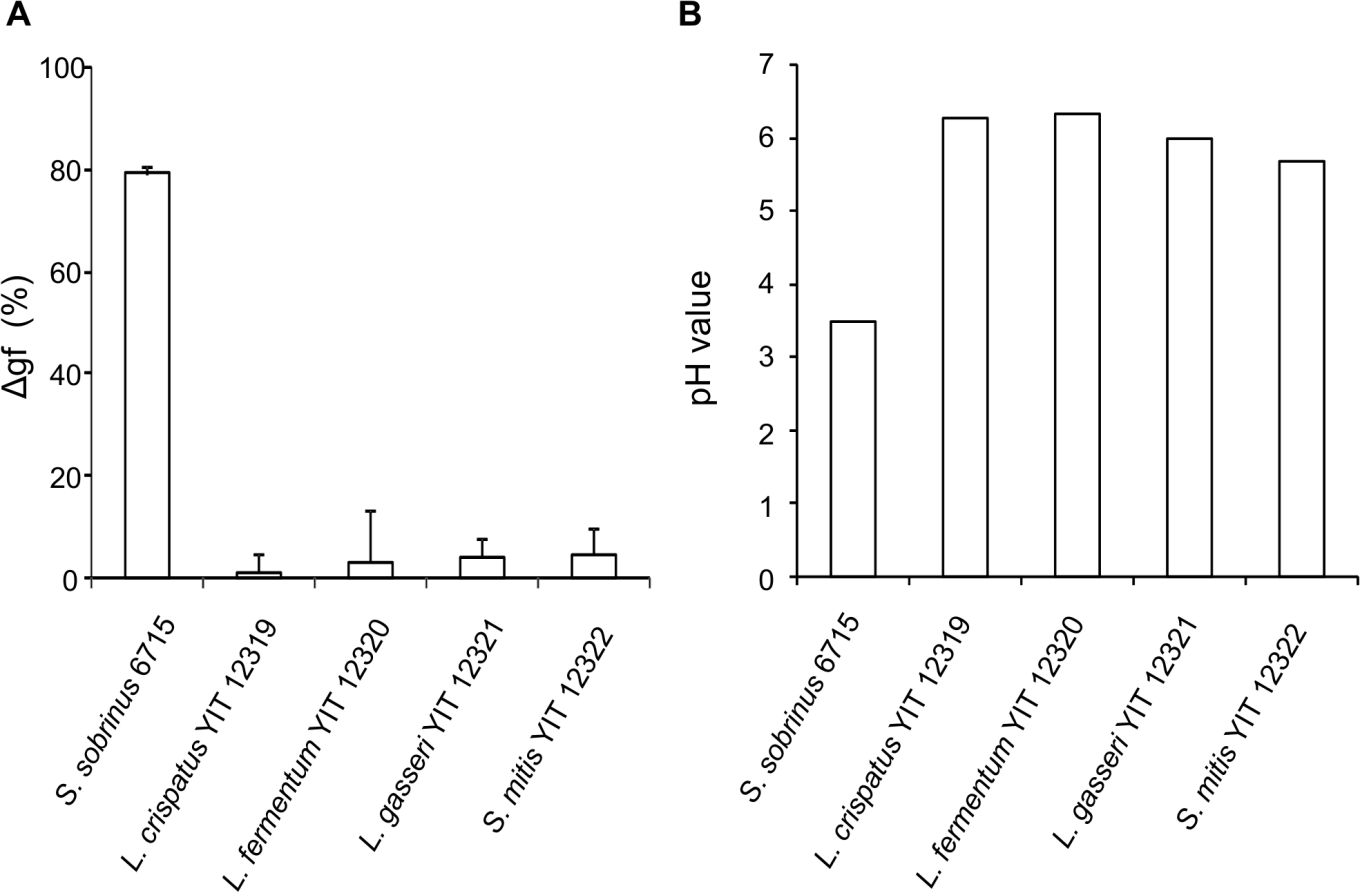
Effects on demineralization of bovine tooth enamel slabs in an AMS. (A) Demineralization was evaluated using the Vicker’s hardness values of the enamel slabs before and after incubation with 4 LAB isolates (3 lactobacilli and 1 streptococcus) and a positive control (*S*. *sobrinus* 6715). Δgf (%) shows the ratio of hardness after/before incubation. Values are mean ± SD (n = 4). (B) Values indicate the pH values underneath the artificial biofilm that formed on the flat bulb of the pH electrode after incubation.

**Fig 5:**
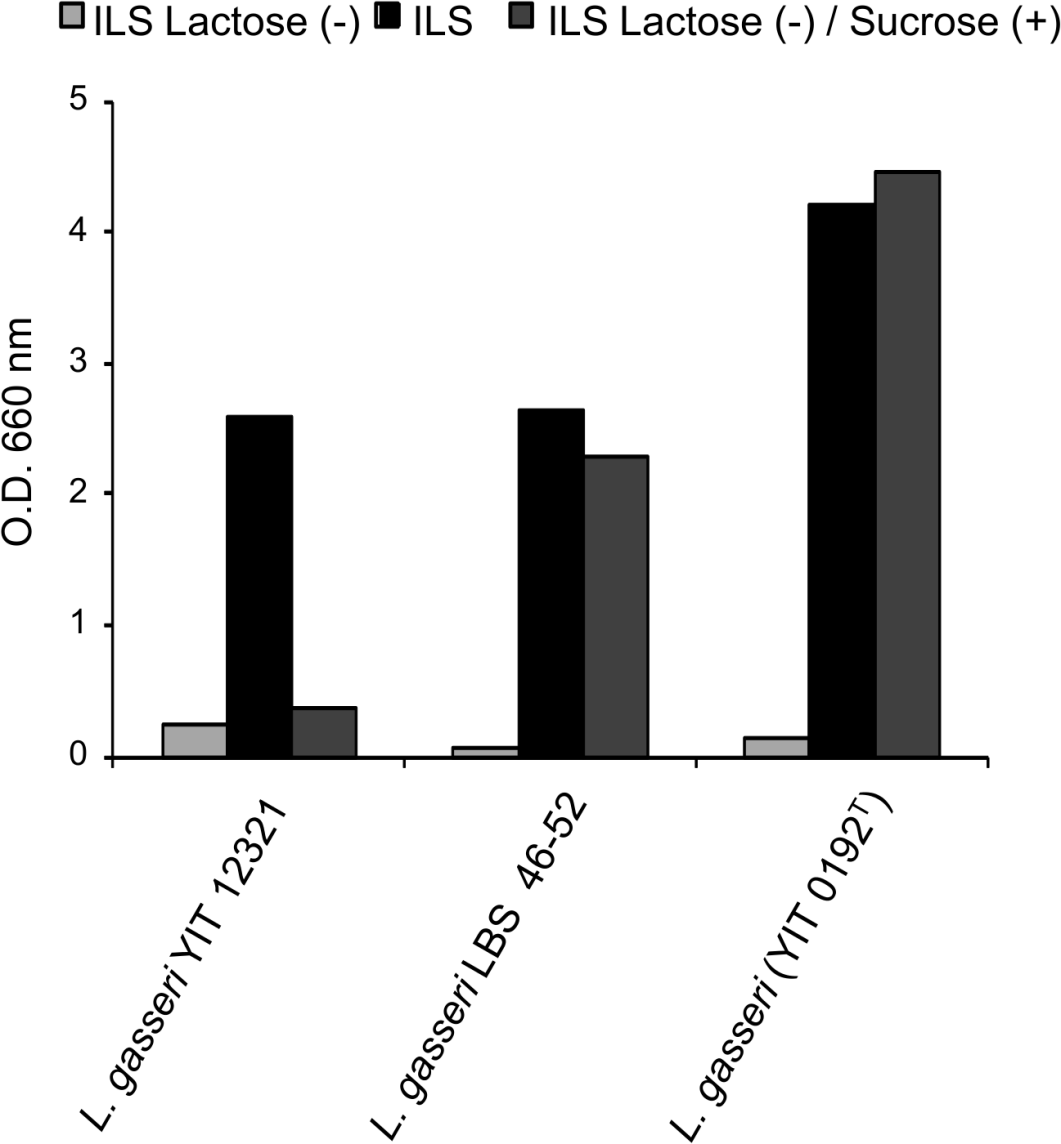
Utilization of sucrose for growth of *L*. *gasseri* YIT 12321. Three strains of *L*. *gasseri* (2 clinical isolates and 1 type strain) grown in 3 different media: ILS broth without lactose, ILS containing lactose, and ILS containing sucrose instead of lactose. The degree of growth of each strain was evaluated by the O.D. 660 nm value.

Fig 4 shows the effects of 4 oral LAB isolates, which were selected from the 5 oral isolates screened, and a positive control on the demineralization of bovine tooth enamel slabs, pH change, and the production of WIG on the enamel in an AMS. After incubation in nutrient medium (MRS/TS) containing 2.5% sucrose (final concentration 1% on the flat pH electrode), *S*. *sobrinus* 6715 decreased the pH to 3.5, reduced the Vicker’s hardness value of the enamel slabs by approximately 80%, and produced WIG, suggesting that the positive control had the potential to demineralize the enamel as the initial step of cariogenicity. All isolates, *L*. *crispatus* YIT 12319, *L*. *fermentum* YIT 12320, *L*. *gasseri* YIT 12321, and *S*. *mitis* YIT 12322, maintained the pH above 6 and showed neither a reduction of the Vicker’s hardness value of the enamel slabs nor WIG production. The utilization of sucrose was evaluated for the 4 isolates in ILS medium containing 2% sucrose as the sole source of sugar. *L*. *gasseri* YIT 12321 did not grow in the medium, showing no utilization of sucrose (Fig 5), but the others utilized sucrose and grew.

## Discussion

This study screened new probiotic candidates with potential oral health benefits and no harmful effects, including cardiovascular disease. We evaluated their production of VSCs, antibacterial activity against pathogenic bacteria causing periodontal disease and dental caries, adherence activity to S-HA or oral epithelial cells *in vitro*, cariogenic potential in an AMS, and risk of experimental infectious endocarditis in a rat model.

The transit time of foods is shorter in the oral cavity than in the other areas of the digestive tract, and oral bacteria are transferred to the stomach together with saliva, which is secreted in large volumes. Therefore, oral probiotics must have the potential to adhere to oral tissues. *S*. *sanguinis*, *S*. *gordonii*, *S*. *oralis*, and *S*. *mitis* are dominant oral species that reportedly adhere to the pellicle of the tooth surface and contribute to the formation of an oral biofilm at the early stage of its development [40]. *S*. *sanguinis* and *S*. *salivarius* also reportedly have adherent pili that contribute to their adherence to mucin [41], a major component of saliva, and to tooth surfaces [2]. In contrast, lactobacilli are generally known to have lower adherence ability to teeth than streptococci. In our screening (Table 3), most of *S*. *salivarius*, *S*. *oralis*, *S*. *parasanguinis*, and *S*. *mitis* strains demonstrated adherence to S-HA, which was used as an alternative to human teeth. Adherence activity was lower in lactobacilli than in streptococci, except for *L*. *fermentum* and *L*. *gasseri*. Our observations are consistent with findings reported previously [41,42].

In the intestine, bacteria do not adhere to the mucosal layer directly because the thick mucin layer acts as a reservoir of microbes. In contrast, oral epithelial tissue is coated with a thin mucosal layer, which consists of mucin from the saliva in the oral cavity. Oral microbes can adhere directly to oral epithelial tissue or teeth via this mucosal layer. Therefore, saliva can function as a reservoir of harmful and beneficial oral microbes via their adherence to tissue [43]. In fact, tongue coating, which contains food residue and many microbes, is one of the sites at which VSCs are produced as a source of oral malodor [44]. Therefore, it is considered important that oral probiotics have a higher adherence potential to oral epithelial tissue, such as the tongue, rather than to teeth. In our screening (Fig 3), *L*. *crispatus* YIT 12319 and LBS 17–11 had higher adherence activity to HSC cells, which are derived from the human tongue. In addition, we classified the bacteria into 3 types on the basis of their adherence activity: isolates showing similar adherence to both HSC and HO cells, and isolates adhering specifically to either HSC cells or HO cells. These results suggest that common and specific receptor-ligand systems may exist between epithelial cells and bacteria. As an intestinal-dwelling species of *L*. *crispatus* reportedly possesses S-layer proteins that facilitate its adherence to epithelial HT-29 cells and Caco-2 cells from the colon [45], oral *L*. *crispatus* is expected to adhere to the epithelial cells of the tongue via S-layer-like proteins.

LAB reportedly show antibacterial activity against periodontal disease pathogens such as *P*. *gingivalis* and *P*. *intermedia* [46]. As *P*. *gingivalis* and *P*. *intermedia* are highly sensitive to acid [47], most of their activity is considered to be due to the lactic acid and other organic acids they produce. Meanwhile, *S*. *sobrinus* and *S*. *mutans*, as cariogenic pathogens, are acid tolerant because they express a proton-ATPase [48]. Recently, it was reported that lactic acid and other organic acids contribute to less than 50% of the antibacterial activity of several LAB [49], and bacteriocins have been isolated from some lactobacilli and streptococci, such as salivaricin from a strain of *L*. *salivarius* [50] and a strain of *S*. *salivarius* K12 [51], reuterin from a strain of *L*. *reuteri* [52], and plantaricin from a strain of *L*. *plantarum* [53]. In our screening (Fig 2), many oral LAB isolates showed antibacterial activity against *P*. *gingivalis* and *A*. *actinomycetemcomitans* after neutralization, suggesting that the LAB may have the potential to produce bacteriocins or other antibacterial substances in the culture supernatant.

VSCs (hydrogen sulfide, methyl mercaptan, and dimethyl sulfide) are produced by periodontal pathogenic bacteria from sulfur compounds such as L-cysteine and L-methionine under anaerobic conditions. A recent study showed that the production of VSCs is associated with the progression of periodontal disease [7]. Therefore, oral probiotics must have no potential to produce VSCs. Six potent VSC producers were found among 46 isolates of oral lactobacilli [23]. In our study (Table 1), 391 isolates from 896 oral isolates with higher VSC producing ability than *S*. *mitis* YIT 2035^T^ were excluded from the screening. For the lactobacilli, *L*. *oris* YIT 0277 (hydrogen sulfide, 5.73 μg/10 mL culture/1 OD_550_; methyl mercaptan, 1.2 μg/10 mL culture/1 OD_550_) and *L*. *paracasei* LBS 26–13 (hydrogen sulfide, 9.65 μg/10 mL culture/1 OD_550_; methyl mercaptan, 1.36 μg/10 mL culture/1 OD_550_) showed a high level of VSC production. *L*. *paracasei* LBS 26–13 was isolated from a healthy subject with no oral malodor. Lactobacilli were detected at approximately 10^5^ CFU/g dental plaque in this healthy subject and estimated at 0.004% of the total number of oral anaerobes (data not shown). It is suggested that oral lactobacilli are a very minor habitant in this subject, whose oral concentration of VSCs was lower than the detection limit. It is also indicated that a potent producer of VSCs can exist endogenously even in healthy subjects, and the control of endogenous harmful bacteria is important to maintain oral health.

Infective endocarditis is inflammation of the inner tissue of the heart valves caused by infectious bacteria, whose attachment to the surface of an abnormal valve triggers the formation of vegetation containing microcolonies of bacteria, immune cells, fibrin, and blood platelets [54]. It has been reported that several species of oral streptococci, such as *S*. *sanguinis*, *S*. *salivarius*, and *S*. *oralis*, are isolated frequently from the vegetation of patients with endocarditis [20], but lactobacilli are isolated very rarely from such patients [21]. In addition, a clinical isolate of *L*. *rhamnosus* reportedly induces experimental infective endocarditis at a dose of 1.0 × 10^6^ CFU in a rat model [55] and a rabbit model [35]. In our screening (Table 4), 4 lactobacillus isolates showed no induction of experimental infective endocarditis at the same dose in a rat model; however, 9 streptococcus isolates did induce infective endocarditis, except for *S*. *mitis* YIT 12322. In further research, we will examine the differences between *S*. *mitis* YIT 12322 and the other streptococci. There are possible factors associated with the induction of infective endocarditis, such as adherence activity to fibrin, laminin, fibronectin, collagen, and blood platelets as component of vegetation and the expression of adherence genes.

Tooth decay remains one of the most common oral diseases worldwide, although the proportion of the elderly population with many teeth is increasing in developed countries due to the development of daily dental care for the improvement of oral health [3]. Tooth decay is initiated by the adherence of early colonizers such as *S*. *oralis*, followed by pathogenic bacteria such as *S*. *sobrinus* and *S*. *mutans* to tooth surfaces to form a dental plaque, which is a biofilm of colonized bacteria with WIG-producing abilities. Thereafter, the demineralization and destruction of enamel are caused by lowering the pH below 5.5 due to the acid produced by the bacteria from sugars in the plaque [56]. Therefore, oral probiotics must have no cariogenic potential, such as enamel demineralization associated with no or low adherence to S-HA, no production of WIG, and maintaining the pH above 5.5. In our screening (Fig 4), *L*. *crispatus* YIT 12319, *L*. *fermentum* YIT 12320, *L*. *gasseri* YIT 12321, and *S*. *mitis* YIT 12322 showed neither enamel demineralization, decrease of pH below 5.5, nor the production of WIG on the enamel following incubation in a 1.0% sucrose solution in an AMS, despite potent acid production from glucose. It is indicated that the lack of adherence to tooth surfaces or S-HA is associated with the absence of primary cariogenic potential in potent acid producers such as *L*. *fermentum* YIT 12320 and probably *L*. *crispatus* YIT 12319. It is also suggested that the no or low acid production from sucrose by *L*. *gasseri* YIT 12321 and *S*. *mitis* YIT 12322 with adherence activity to S-HA could be a reason for their lack of primary cariogenic potential.

In conclusion, this study demonstrates that 4 oral isolates from healthy subjects, *L*. *crispatus* YIT 12319, *L*. *fermentum* YIT 12320, *L*. *gasseri* YIT 12321, and *S*. *mitis* YIT 12322 (Table 5), are considered as new probiotic candidates because of their lack of VSC production, higher antibacterial activity against some oral pathogenic bacteria, higher adherence activity to oral epithelial cells *in vitro*, no potential of primitive cariogenicity, such as no demineralization of tooth enamel or production of WIG on the enamel in the AMS, and a lower risk of experimental infectious endocarditis in a rat model. These candidates are expected as new probiotics with potential oral health benefits and no harmful effects.

**Table 5:** List of probiotic candidates selected from oral LAB isolates.

Strain No.	Antibacterial activity against oral pathogenic bacteria^a^ ^,^ ^b^												Utilization of sucrose	Adherence activity to		
	*Pg*		*Pi*		*Aa*		*Sm*		*Ss*		Total (mm)			S-HA^c^	HO cells^d^	HSC cells^e^
	(−)	(+)	(−)	(+)	(−)	(+)	(−)	(+)	(−)	(+)	(−)	(+)			(cells/1.0 mm^2^)	
***L*. *crispatus* YIT 12319**	4.7	6.1	5.2	0.0	2.0	3.0	0.3	0.0	0.8	0.0	13.0	9.1	+	N.D.	66.7	864.6
***L*. *fermentum* YIT 12320**	5.4	2.6	4.5	0.0	4.5	0.0	0.0	0.0	0.0	0.0	14.3	2.6	+	-	93.75	265.63
***L*. *gasseri* YIT12321**	3.5	3.6	2.2	0.0	0.0	0.0	0.0	0.0	0.0	0.0	5.7	3.6	-	+	41.7	169.8
***S*. *mitis* YIT 12322**	3.2	4.6	4.5	-	0.0	0.0	0.0	0.0	0.0	0.0	7.7	4.6	+	+	128.1	72.9

^a^ *Pg*, *P*. *gingivalis* ATCC 33277; *Pi*, *P*. *intermedia* ATCC 25611; *Sm*, *S*. *mutans* ATCC 25175; *Ss*, *S*. *sobrinus* ATCC 33478; *Aa*, *A*. *actinomycetemcomitans* Y4.

^b^(−); Without neutralization, (+); with neutralization.

^c^S-HA; salivary-coated hydroxylapatite.

^d^Cells originating from human buccal mucosa carcinoma.

^e^Cells originating from human tongue carcinoma.

Further studies are necessary to establish a probiotic strain showing neither the potential to propagate antibiotic resistance, general and genetic toxicity in animal models, nor side-effects during human studies. After the safety of candidate probiotics for humans is examined in animal and human studies (Phase 1), it is necessary to examine whether the selected oral probiotic candidates can remain in the human oral cavity to decrease the levels of oral pathogenic bacteria, and whether these candidates have an effect and are safe as probiotics in human clinical trials (Phase 2). At the same time, it is also necessary to examine what product form, such as tablets or dairy products, and what amount of intake is sufficient to enhance their retention in the oral cavity.
